# A roadmap for the genetic analysis of renal aging

**DOI:** 10.1111/acel.12378

**Published:** 2015-07-29

**Authors:** Gerda A Noordmans, Jan-Luuk Hillebrands, Harry van Goor, Ron Korstanje

**Affiliations:** 1 Department of Pathology and Medical Biology, University of Groningen, University Medical Center GroningenGroningen, the Netherlands; 2 The Jackson LaboratoryBar Harbor, ME, USA

**Keywords:** aging, genetics, human, kidney, mouse, phenotype

## Abstract

Several studies show evidence for the genetic basis of renal disease, which renders some individuals more prone than others to accelerated renal aging. Studying the genetics of renal aging can help us to identify genes involved in this process and to unravel the underlying pathways. First, this opinion article will give an overview of the phenotypes that can be observed in age-related kidney disease. Accurate phenotyping is essential in performing genetic analysis. For kidney aging, this could include both functional and structural changes. Subsequently, this article reviews the studies that report on candidate genes associated with renal aging in humans and mice. Several loci or candidate genes have been found associated with kidney disease, but identification of the specific genetic variants involved has proven to be difficult. *CUBN, UMOD,* and *SHROOM3* were identified by human GWAS as being associated with albuminuria, kidney function, and chronic kidney disease (CKD). These are promising examples of genes that could be involved in renal aging, and were further mechanistically evaluated in animal models. Eventually, we will provide approaches for performing genetic analysis. We should leverage the power of mouse models, as testing in humans is limited. Mouse and other animal models can be used to explain the underlying biological mechanisms of genes and loci identified by human GWAS. Furthermore, mouse models can be used to identify genetic variants associated with age-associated histological changes, of which *Far2, Wisp2,* and *Esrrg* are examples. A new outbred mouse population with high genetic diversity will facilitate the identification of genes associated with renal aging by enabling high-resolution genetic mapping while also allowing the control of environmental factors, and by enabling access to renal tissues at specific time points for histology, proteomics, and gene expression.

## Introduction

Aging of the kidney is associated with structural changes and functional decline, which renders the elderly more vulnerable to superimposed stress factors such as heart failure, dehydration, hypertension, and eventually the evolution of chronic kidney disease (Miller, 1995; Ungar *et al*., 2000). The geriatric population is increasing, making chronic kidney disease an important global public health problem with a high prevalence and morbidity (Levey *et al*., 2007). Transient renal conditions, exposure to chronic diseases with altered metabolic states, and certain diets or drugs predispose to accelerated renal aging. In addition, several studies show evidence for the genetic basis of renal disease (summarized in Garrett *et al*., 2010). In order to determine why some elderly individuals are more prone than others to the development of renal damage, it is important to identify the genes associated with this susceptibility. To accomplish this, a clear definition of the renal aging phenotype is essential for the interpretation of genetic studies. Such a phenotype includes structural changes such as glomerulosclerosis and tubular atrophy, and functional changes including albuminuria and decreased glomerular filtration rate (GFR).

The purpose of this opinion article was to provide a roadmap of approaches for performing genetic analyses leading to identification of the genes involved in renal aging. First, an overview of the phenotypes associated with renal aging is given to differentiate between the phenotypes used in studies of non-age-related kidney disease and those used in studies of age-related kidney disease. Based on these aging-related phenotypes, an overview of the studies performed to date that report on loci or candidate genes associated with renal aging in humans and mice, and the possible roles of some of these genes, is discussed. Finally, we provide suggestions on how to move forward in order to better understand the underlying pathways of renal aging and to unravel the process in more detail in the near future.

## Phenotypes of renal aging

Phenotypic changes in renal aging include both functional and structural changes, but none of these features are pathognomonic for aging. Aging is a multifactorial process, and it is difficult to differentiate between changes due to aging and those due to diseases that affect the aging kidney (Zhou *et al*., 2008). We should keep in mind that accelerated renal aging could also reflect accelerated aging overall. For example, cardiovascular aging can lead to hypertension, which can increase the rate of kidney aging. However, in this study, we will focus on the aging phenotypes of the kidney. Furthermore, to perform genetic analysis and accurately identify the genes associated, precise phenotyping is crucial. The structural and functional phenotypes of the aging kidney will be discussed. However, it is important to recognize that these structural and functional parameters may not yield the ‘right aging phenotype’ on which we should base our genetic analysis.

### Functional changes

Decline in GFR is one of the primary functional changes seen with aging. An interesting overview of studies that report on renal function decline in the aging population has recently been published (Bolignano *et al*., 2014). The severity of decline in these studies ranges from 0.4 to 2.6 mL min^−1^ annually; however, in a third of the elderly, the GFR remains constant. In the KDIGO guidelines, renal function impairment is set at a GFR <60 mL min^−1^, which results in chronic kidney disease (CKD) in 47% of adults aged 70 years and older (Levey *et al*., 2005; Coresh *et al*., 2007). As almost half of the elderly meet the CKD criteria, it is questionable whether a CKD diagnosis is relevant in this age group, or whether this GFR decline should be seen as a process of normal aging (Poggio *et al*., 2009; Schaeffner *et al*., 2012).

Few data exist on normal kidney function in the elderly. Our knowledge of renal function decline in the aging population is disputable, as it is based on equations that are not validated in an aged population, and on the use of serum creatinine for these equations. Formulas using serum creatinine to estimate GFR are unreliable in the elderly, as it is influenced by nutritional status and muscle mass. Several equations have been developed to estimate GFR, such as the Cockcroft–Gault (CG), modification of diet in renal disease (MDRD), and CKD-EPI creatinine/cystatin C, but none has been validated in an older population. As a result, in 40% of the very elderly (≥80 years of age), GFR estimations by the CG and MDRD differ by over 30% (Van Pottelbergh *et al*., 2011). While the use of an equation incorporating a combination of creatinine and cystatin C showed improved accuracy, the use of cystatin C is costly and is influenced by inflammation (Hallan & Gansevoort, 2013). Based on the above evidence, it is important to reference any estimated GFR (eGFR) value to age and gender percentiles, and to use specifically adapted eGFR equations with the elderly. As it is questionable whether any creatinine-based equation is sufficiently precise in the elderly, it is doubtful whether the eGFR would be a good value on which to base genetic studies of age-related kidney disease, at least until we have age-specific reference data. Better parameters that can be used for precise phenotyping to identify the genes involved in kidney aging include albuminuria and abnormal histology, the latter of which is at the basis of functional changes.

Albuminuria is also included in the staging system for CKD, defined as an albumin-to-creatinine ratio >30 mg g^−1^ (Levey *et al*., 2005). Albuminuria is a strong predictor of cardiovascular disease, especially in patients with diabetes and hypertension (Brantsma *et al*., 2006; de Jong & Curhan, 2006). This suggests that albuminuria might be the manifestation of a systematic disturbance of the renal microvasculature and a disturbance of endothelial cell function. Therefore, microalbuminuria with a normal eGFR might be a presentation of a systemic disturbance affecting all microcirculations, including the renal microvasculature. However, albuminuria was also found to be an early sign of progressive renal function loss in a nondiabetic population, showing an association between micro-albuminuria (30–300 mg per day) and glomerular hyperfiltration (Pinto-Sietsma *et al*., 2000). In the aging population, there is an increase in the percentage of sclerotic glomeruli, with compensatory glomerular enlargement and hyperfiltration; thus, it is plausible that albuminuria may also be an indicator of glomerular hyperfiltration due to structural changes in aging individuals. As we cannot be sure whether albuminuria is the consequence of microvascular changes induced by underlying disease or results from aging, we should either exclude patients with confounding factors such as diabetes or hypertension from our study population or correct for these.

### Structural changes

**Fig 1:**
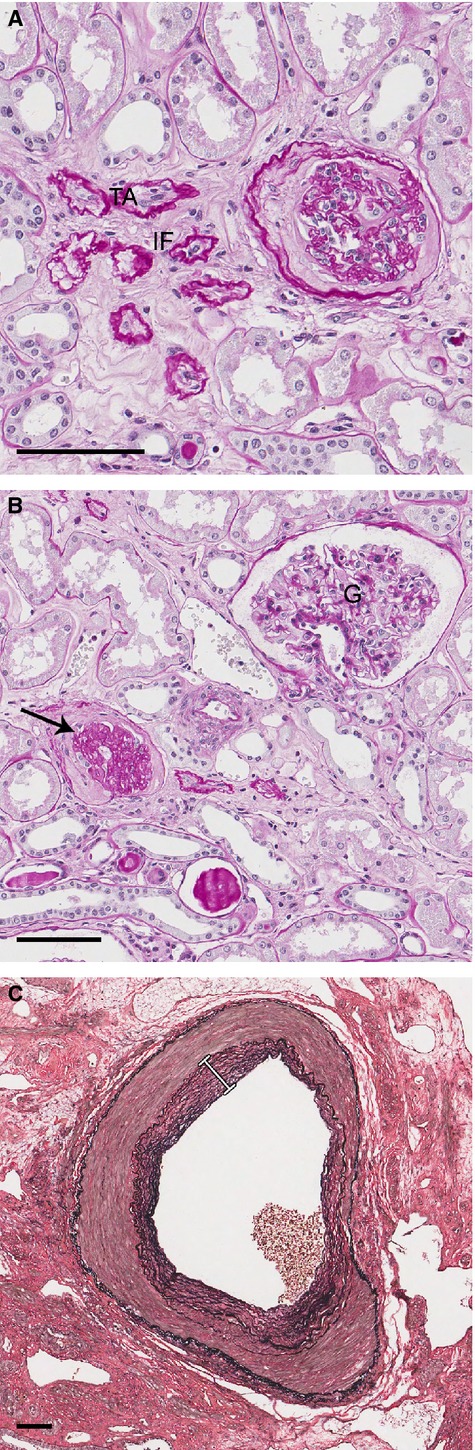
Structural changes of the human aging kidney. (A). Interstitial fibrosis (IF) and tubular atrophy (TA) (PAS, scale bar, 100 μm). (B). Glomerulus (G), glomerulosclerosis (arrow) (PAS, scale bar, 100 μm). (C). Intima fibrosis of a renal interlobar artery (white line) (Verhoeff’s stain, scale bar, 100 μm).

After middle age in adults, kidney mass decreases progressively; this reduction is more pronounced in the cortex than in the medulla and can reach up to 40% in patients in their eighties (Gourtsoyiannis *et al*., 1990; Hoy *et al*., 2003; Zhou *et al*., 2008). Tubular-interstitial changes related to kidney aging include increased tubular atrophy with surrounding areas of interstitial fibrosis (Fig.1A), which could explain the age-related reduction in kidney mass (Martin & Sheaff, 2007; Rule *et al*., 2010).

The number of functioning glomeruli decreases throughout adult life, while the percentage of sclerosed glomeruli increases (Fig.1B). After the age of 40, the percentage of sclerotic glomeruli in the normal population exceeds 10% (Kaplan *et al*., 1975; Hoy *et al*., 2003; Rule *et al*., 2010). Furthermore, with advancing age, a decrease in glomerular density is seen. Interestingly, in a human biopsy study, an increase in glomerular density with age was seen when the percentage of sclerotic glomeruli was >10% (Rule *et al*., 2011). Possible explanations for this finding were a higher prevalence in older donors of tubular atrophy, which is a lesion associated with loss of kidney volume, and the fact that the compensatory mechanism for glomerulosclerosis – hypertrophy of the unaffected nephrons – is reduced in older kidneys. Older age is indeed characterized by glomerular enlargement, especially in the superficial cortex. This compensatory hypertrophy of functional glomeruli may possibly develop as a response to an increased percentage of sclerotic glomeruli (McLachlan, 1978; Samuel *et al*., 2005). To accommodate glomerular enlargement with advancing age, it is known from rat studies that podocytes increase in size instead of number, but eventually a certain threshold is reached and podocyte hypertrophy decompensates with widening of the foot processes, decreased filtering efficiency, proteinuria, and eventually podocyte depletion, all of which result in matrix accumulation and glomerulosclerosis (Wiggins *et al*., 2005; Wiggins, 2012).

Aging of the glomerulus is also associated with thickening of the glomerular basement membrane, mesangial expansion, and eventually global sclerosis (Fig.1B), although these features can also be found in other renal disorders, such as focal segmental glomerulosclerosis (FSGS) and diabetic nephropathy, and can result from chronic inflammation, hypertension, or vascular disease (Anderson & Brenner, 1986; Zhou *et al*., 2008; Wiggins, 2012). Glomerular membrane thickening was found to be the pathological parameter most strongly correlated with chronological aging of the kidney in a study of aging in five organs throughout the entire lifespan of a C57BL/6J female mouse (Jonker *et al*., 2013). Vascular changes seen with kidney aging include intima fibrosis and medial hypertrophy (Fig.1C) (McLachlan, 1978).

Overall, the prevalence of nephrosclerosis, which includes glomerulosclerosis, tubular atrophy, interstitial fibrosis, and arteriosclerosis (Fig.1), was found to increase with age in healthy adults. Interestingly, this increase could not be explained by age-related differences in kidney function or CKD risk factors, and thus, a renal biopsy is the only clinical test for detecting this subclinical age-related nephropathy (Rule *et al*., 2010). Hypertension, nocturnal blood pressure, and urine albumin were also found to be associated with an increased prevalence of nephrosclerosis, independent of age. Although studies indicate that these characteristics can have renal damage as the underlying cause, we cannot be sure whether these characteristics are the cause or consequence of morphological changes that we expect to observe in kidney aging (Johnson *et al*., 2002; Brantsma *et al*., 2006).

To conclude, precise phenotyping is crucial if we want to perform genetic analysis of renal aging. Phenotypic changes associated with renal aging include functional and structural changes. Equations used to date to estimate glomerular filtration rate are imprecise for the elderly population and therefore are not the optimal parameter for phenotyping of renal aging. Albuminuria due to glomerular enlargement and hyperfiltration is a good indicator of renal aging, as long as we exclude or correct for confounding factors. Structural changes such as glomerulosclerosis, interstitial fibrosis, tubular atrophy, and arteriosclerosis are good indicators of renal aging, if captured in the early stage of their evolution and in the absence of confounding kidney disease. These histological changes can be detected before they actually affect kidney function.

## Studies to date of genes involved in age-related kidney disease

### Human

Familial aggregation studies and segregation analyses provided evidence for the genetic basis of renal disease (Garrett *et al*., 2010). Thereafter, efforts have been made to identify genes that contribute to renal disease by performing candidate gene analyses and genome screen analyses, the latter of which has the capacity to identify novel genes.

Genetic analysis of several kidney disease phenotypes has been performed, including focal segmental glomerulosclerosis (FSGS), end-stage renal disease (ESRD; GFR<15 mL min^−1^, requiring renal replacement therapy), albuminuria, and high serum creatine levels and estimated glomerular filtration rates (eGFRs) (Fox *et al*., 2004; Chen *et al*., 2007; Kopp *et al*., 2008; Kottgen *et al*., 2009, 2010; Pattaro *et al*., 2009, 2012; Genovese *et al*., 2010a,b; Boger *et al*., 2011; Parsa *et al*., 2013). Only a few of these studies may be relevant to investigation of the genetics of renal aging, as many used a middle-aged study population. In our opinion, the study population should comprise individuals at least over 70 years of age, because at this age, more than half of the elderly show age-related histological changes (Rule *et al*., 2010). Most genetic studies of human kidney disease phenotypes focus on functional changes, and lack structural data or are performed using a cohort with other confounding factors, which makes it difficult to determine whether the identified genomic regions could be involved in renal aging or are associated with other risk factors.

Böger *et al*. performed a meta-analysis in a large European population in which they identified a missense SNP in the *CUBN* gene that was associated with albuminuria (Boger *et al*., 2011). The mean age of the study group in which the SNP in *CUBN* was found to be associated with albuminuria ranged from 42 to 74 years. As this association was also found in the general population, independent of risk factors such as hypertension and diabetes, renal aging might play a role, although the authors do not report biopsy data. A focus on the possible function of *CUBN* in renal aging will be discussed in the next section.

Several genomewide association studies (GWAS) of different measures of kidney function have been performed. Pattaro *et al*. (2009) performed genomewide linkage analysis of serum creatinine on three isolated populations, observing a higher heritability when excluding diabetics and individuals on antihypertensive treatment, proving substantial heritability of serum creatinine levels in healthy individuals. Linkage to chromosome 22q13 was detected, containing the *MYH9* gene, which was previously linked to other pathological renal conditions (Pattaro *et al*. (2009). However, as the mean age of this study population was 49 years, and the study examined serum creatinine levels, we cannot extrapolate results from this study to renal aging.

The CKDGen consortium has conducted several GWAS and identified various loci and genes associated with CKD and kidney function. The first study was in four European population-based cohorts and identified susceptibility variants for renal function and CKD at the *UMOD*, *SHROOM3*, and *SCT1* loci (Kottgen *et al*., 2009). Another GWAS in a larger study group additionally identified 13 new loci in genes related to nephrogenesis, glomerular filtration barrier formation, podocyte function, and angiogenesis (Kottgen *et al*., 2010). An additional study in a large European population was performed to identify genes that influence kidney function differently depending on the following CKD risk factors: sex, age, hypertension, and diabetes. Six new loci were found to be associated with eGFR, among which *UMOD* clearly had a stronger association in older individuals (Pattaro *et al*., 2012). Recently, the CDKGen consortium performed a GWAS that analyzed kidney function decline over time instead of cross-sectional eGFR (Gorski *et al*., 2015). The *UMOD* locus was again found to be associated with kidney function decline. In addition, the *GALNT11* and *CDH23* loci were suggested to play a role in deterioration of kidney function. Briefly, the CKDGen consortium has identified various genes associated with CKD and kidney function, of which the mechanistic function of *UMOD* and *SHROOM3* has been studied in the greatest detail, which will be discussed in the following section. The CDKGen consortium uses large cohorts with a broad range in mean age at baseline between the different cohorts and a variety in duration of follow-up as well, which gives the possibility to study the age-related changes in kidney function. However, eGFR was used for phenotyping and there are no biopsy data available to study structural changes in the different cohorts.

There are a few studies that looked specifically into the genetics of renal aging in humans. A whole-genome analysis of gene expression as a function of age was performed in kidney samples from patients aged 27–92 years (Rodwell *et al*., 2004). Over 900 age-dependent genes were found, with a similar aging profile for the cortex and medulla. This work showed that aging probably involves an accumulation of small changes in expression from many genes, rather than the increased expression of only a few genes. In another study, cDNA microarrays were used to study gene-expression profiles of human kidneys between three different age groups (Melk *et al*., 2005). About 500 genes were differentially expressed between these age groups, with the kidneys of elderly individuals overexpressing genes associated with extracellular matrix synthesis and low-grade focal inflammation.

A similar strategy was used by Wheeler *et al*. (2009) by looking at transcriptional profiling, but adding expression quantitative trait loci (eQTL) mapping afterward to determine which of the age-regulated genes contain SNPs that associate with expression level. An SNP in *MMP20*, a matrix metalloproteinase, was associated with kidney aging, explaining 1%-2% of the variance in GFR among individuals.

These studies show that many genes probably change expression during aging. Although these data give an idea about which mechanisms are involved, it does not determine the exact underlying biological pathways, which would provide the opportunity to intervene in the future. This approach gives only a general view of changes in expression with aging, most likely due to the lack of specific phenotyping.

### Possible role of candidate genes in the aging process explained using animal models

As described in the previous section, human GWA studies identified several candidate genes associated with the functional parameters albuminuria and eGFR. For some of these genes, additional evidence points to aging as an important variable, which will be discussed in more detail (Table1). This discussion illustrates the ways in which animal models can be used to further explore the biological function of genes identified by human GWAS.

**Table 1:** Candidate genes involved in renal aging

Gene	Analyzed phenotype	Possible function in renal aging
*CUBN*	Albuminuria	Cubilin functions as an endocytic receptor of albumin. A defect in this tubular reabsorption leads to albuminuria.
*UMOD*	CKD, eGFR decline	UMOD influences NaCl reabsorption; overexpression leads to salt-sensitive hypertension.
		Overexpression also causes age-dependent lesions; tubular casts and tubular dilatation.
*SHROOM3*	CKD	A defect in the actin-binding domain of SHROOM3 results in impairment of the glomerular filtration barrier leading to albuminuria and glomerulosclerosis.
		Increased SHROOM3 expression is associated with fibrosis.
*Far2*	Mesangial matrix expansion	Overexpression of *Far2* leads to upregulation of PAF and TGFβ; profibrotic factors.
*Wisp2*	Tertiary lymphoid organs	Wisp2 is part of the Wnt signaling pathway and linked to aging-related inflammation
*Esrrg*	Glomerular lipoprotein depositsTertiary lymphoid organs	This orphan nuclear receptor influences lipid metabolism, is involved in cardiovascular disease, and associated with altered blood pressure.
		Exact mechanism unknown.

*CUBN* encodes the protein cubilin, which is expressed in the apical brush border of proximal tubular cells (Christensen & Birn, 2002). Cubilin functions as an endocytic receptor of albumin via interaction with megalin. *CUBN* is involved in age-related albuminuria due to a defect in tubular reabsorption. The age-related changes of the megalin/cubilin interaction were studied in rats in which a decrease in the amount of megalin was observed with age, and although the levels of the native cubilin form did not change expression, the levels of fragmented cubilins significantly increased in aged rats (Odera *et al*., 2007). These cubilin fragments might be the consequence of degeneration of tubular cells via accumulation of albumin. The accumulation of fragmented cubilin might further promote albuminuria if the fragmented cubilin still interacts with megalin while being unable to bind albumin. However, the genetic variance in *CUBN* that leads to albuminuria is still unknown. Interestingly, in the intestine of mice, only a limited number of genes were found to be differently expressed with aging, among which *CUBN* showed decreased expression, likely influencing aging-induced vitamin B12 deficiency (Steegenga *et al*., 2012). From these results, we can conclude that *CUBN* is associated with aging. In the kidney, it is associated with age-related albuminuria via a defect in tubular reabsorption. However, it is still unknown which genetic variant in *CUBN* leads to or predisposes for albuminuria.

Several GWA studies identified *UMOD* as being associated with eGFR and CKD (eGFRcrea <60 mL min^−1^) (Kottgen *et al*., 2009, 2010; Pattaro *et al*., 2012; Gorski *et al*., 2015). The association with eGFR is stronger in individuals >65 years of age, which might indicate an important role for *UMOD* in age-associated renal function decline. *UMOD* encodes uromodulin, also known as Tamm-Horsfall protein, which is expressed in the thick ascending limb (TAL) and secreted abundantly in normal urine (Scolari *et al*., 2014). UMOD seems to play a role in inflammatory reactions, having a protective effect against urinary tract infections. However, the exact mechanism of how uromodulin is involved in the immune response has not been elucidated. Furthermore, UMOD influences NaCl reabsorption by the TAL through regulation of the sodium potassium-chloride cotransporter (NKCC2) and the renal outer medullary potassium (ROMK) channel. This regulatory function explains the fact that overexpression of uromodulin in transgenic mice caused salt-sensitive hypertension, which further increased with age (Trudu *et al*., 2013). Overexpression of UMOD also caused age-dependent renal lesions with tubular dilatation and increased tubular cast area. These histological changes can also be observed in individuals above 65 years of age, with increased severity in individuals homozygous for *UMOD* risk alleles (Trudu *et al*., 2013).

To put it concisely, *UMOD* likely has an important role in age-related renal function decline because a stronger association with renal function decline in older individuals was observed; an age-related increase in salt-sensitive hypertension is observed in transgenic mice; and histological changes can be observed in older individuals, with increased severity of the changes in individuals with *UMOD* risk alleles.

Human GWAS also identified *SHROOM3,* which encodes an actin-binding protein, as being associated with CKD (Kottgen *et al*., 2009, 2010; Boger *et al*., 2011). Recently, the biological role of *SHROOM3* was studied in zebra fish and rat models. These studies demonstrated that a defect in the actin-binding domain of SHROOM3 can result in podocyte effacement and impairment of the glomerular filtration barrier, and eventually leads to albuminuria and glomerulosclerosis (Yeo *et al*., 2015). Furthermore, a study of chronic allograft nephropathy showed that the presence of the *SHROOM3* SNP rs17319721 in the donor leads to increased *SHROOM3* expression in allografts and is correlated with increased allograft fibrosis and reduced eGFR (Menon *et al*., 2015). A change in *SHROOM3* expression in the kidney with age has not been reported, but a significant increase with age in human female adipose tissue was observed (Glass *et al*., 2013). In summary, *SHROOM3* has been found to be associated with CKD. Mechanistic evaluation in animal models shows a role for this actin-binding protein in podocyte function and that a risk allele in *SHROOM3* correlates with increased *SHROOM3* expression and fibrosis in allografts. *SHROOM3* may play an important role in renal aging by inducing fibrosis.

Animal models are valuable for functional validation of the genes identified in human GWAS, as testing in humans is limited. *CUBN, UMOD,* and *SHROOM3* are good examples of how animal models can be used for functional analysis of such genes. Despite the identification of a few interesting genes that potentially play important roles in renal aging, we can improve our identification methods using improved diagnostic parameters to analyze age-related changes of the kidney. Phenotyping of renal aging would benefit from the use of histological data. However, because of the invasive nature of the biopsy procedure, it is difficult to obtain kidney tissue in aging individuals for large population studies. Therefore, animal models should be used not only to further investigate the role of genes associated with the pathophysiology of renal aging but also to characterize histological changes in renal aging and to identify causative genes.

### Mouse studies for genetic analysis

Mouse studies are ideal for this purpose, as they are time- and cost-effective; they share 99% of their genes with humans; and the mouse is an excellent model for studying kidney function and damage. Furthermore, mouse models make it possible to control confounding environmental factors and to investigate the most informative phenotypes of renal aging by analyzing the histological changes in detail, and ultimately they provide a platform for studying the biological function of specific candidate genes via experimental intervention studies. Nevertheless, mouse models are still underutilized for the genetic dissection of renal disease and renal aging. Several linkage studies performed in mice have been successful in identifying genomic regions, narrowing quantitative trait loci (QTLs), and comparing QTL regions across species (Doorenbos *et al*., 2008; Hageman *et al*., 2011). However, these studies were performed in young mice (8–10 weeks).

The first genetic analysis of renal phenotypes in aged mice was performed by The Jackson Laboratory’s Nathan Shock Center of Excellence for the Basic Biology of Aging (JAX NSC). In a large group of inbred mouse strains, haplotype association mapping was performed for albuminuria measured at 12, 18, and 24 months of age (Tsaih *et al*., 2010). One significant locus and eight suggestive loci were found, of which some map to previously identified loci associated with albuminuria in the mouse, but with narrowing of the chromosomal location. Two of the nine loci were associated with diabetic nephropathy in humans. The haplotype association mapping technique used on histological phenotypes in the same inbred strains identified *Far2* as being associated with mesangial matrix expansion (MME) in aging mice (Noordmans *et al*., 2013). MME is a characteristic of glomerular aging and a precursor of glomerulosclerosis. This study revealed a 9-bp insertion in the 5’UTR of *Far2* to be associated with the presence of MME and to be absent in most of the strains without MME. *Far2* encodes the protein fatty acyl-coenzyme A reductase 2, which catalyzes the reduction of fatty acyl-CoA to fatty alcohols, a precursor of platelet-activating factor (PAF). Both *in vivo* and *in vitro*, this insertion was associated with increased expression of *Far2*, leading to upregulation of PAF and TGFβ, which are involved in the pathogenesis of sclerosis (Noordmans *et al*., 2013). There were some strains in this study with the *Far2* insertion but in which MME was not observed suggesting additional genes involved in this phenotype.

In conclusion, genetic analysis of a large group of inbred strains identified the gene *Far2* as being associated with age-related mesangial matrix expansion, identifying an insertion of 9 bp as a likely causative variant. The association of *Far2* variants with renal aging in humans has not yet been reported; however, decreased *Far2* expression with age was observed in human skin (Glass *et al*., 2013).

A study of perivascular immune cell clusters and tertiary lymphoid organs (TLOs) in the aged kidneys of the same group of inbred strains as in the previous study identified associations with loci on Chr 1, Chr 2, Chr 8 and Chr 14 in male mice, which included the candidate genes *Esrrg* and *Wisp2* (Huang *et al*., 2014). *Wisp2* is part of the Wnt signaling pathway, and we suggest that a Q-allele in exon 4 of *Wisp2* can promote the development of immune cell clusters, because all strains with a significant cluster size have this allele. However, four strains have the risk allele but showed no perivascular cell clusters, and in this case, other genomic loci could be involved.

In addition, in the same group of inbred strains as those used in the study above, an interesting age-related phenotype of intracapillary glomerular lipoprotein deposits was found to be associated with a 30-kb haplotype block on Chr 1 (Noordmans *et al*., 2014). The region spanning this haplotype block also contained the candidate gene *Esrrg,* but coding or expression differences were not observed between the strains with and without glomerular deposits. *Esrrg* encodes estrogen-related receptor gamma, which is an orphan nuclear receptor expressed in tissues with high metabolic activity, such as the heart and kidney, and is involved in lipid metabolism (Giguere, 2008). *Esrrg* is essential for survival, as knockout mice die perinatally due to cardiomyopathy (Alaynick *et al*., 2007). In humans, *Esrrg* appears to play a role in blood pressure regulation during pregnancy, and a correlation between particular SNPs and altered blood pressure was found (Alaynick *et al*., 2010; Luo *et al*., 2014). Furthermore, a study by Kim *et al*. shows evidence that *Esrrg* is a regulator of hepatic gluconeogenesis (Kim *et al*., 2012). This observation is in line with GWAS data for type II diabetes mellitus showing an association with *Esrrg* variants (Beck *et al*., 2014). In addition, in a study in which 447 genes were identified to have altered expression in the kidney with age, *Esrrg* showed a 1.4-fold decrease with age (Rodwell *et al*., 2004). There is a clear link between *Esrrg* and kidney aging via cardiovascular disease, lipid metabolism, or kidney function itself, but further study is needed to understand the exact mechanism.

## Discussion and future perspectives

An advantage of human populations for genetic studies is that they have a high degree of genetic diversity among individuals. However, with such populations, it is difficult to control potentially confounding factors. An advantage of the inbred strains used in the above studies is the ability to control confounding factors such as environment and population structure. However, using classic inbred strains is at the cost of genetic diversity, because the classic inbred strains are derived from the same subspecies of *Mus musculus* and are related. So important genes might be missed because there is no genetic variation in these genes among the inbred strains. Recently, a high-resolution genetic mapping system was developed using Collaborative Cross (CC) and Diversity Outbred (DO) mice. These two populations are bred from the same eight inbred founder strains, including five classical strains: A/J, C57BL/6J, 129S1/SvlmJ, NOD/ShiLtJ, and NZO/HILtJ; and three wild-derived strains representing different *Mus musculus* subspecies: *Mus musculus musculus, M. musculus domesticus, and M. musculus casteneus*, with more than 45 million SNPs segregating in this population (Collaborative Cross Consortium, 2012, Svenson *et al*., 2012). The DO population is derived from partially inbred CC lines, but is maintained outbred through a random breeding strategy, which makes this population a resource for high-resolution mapping. The mapping resolution is increased below 1 Mb compared to 10–50 Mb for classical genetic crosses. One of the primary advantages of the DO mice over traditional crosses is the ability to rapidly move from a significant QTL to one or a few strong candidate genes. Another major advantage is that the complete genome sequence of all eight founder strains has been determined, which makes causal variant identification possible. The DO mice are a nice complementary resource to the CC lines. Each DO animal is unique, which provides a highly recombinant population with a wide phenotypic variation. CC lines contain the same allelic variants in the reproducible inbred strains, which can be used for mechanistic studies and for predictive validation of mapping results obtained with DO mice.

Focusing on renal aging by studying phenotypic changes, preferably histological changes at the beginning of the disease cascade as described in this review, in the CC lines and DO mice will undoubtedly bring us closer to unraveling the process of renal aging. These DO mice and CC lines might be an interesting tool in addition to human GWAS for identifying and mechanistically evaluating the genes underlying renal aging. For example, characterizing albuminuria in these mice and then performing genetic analysis in mice might enable identification of the causative variant for age-related albuminuria in *CUBN*. Similarly, the quantification of tubular dilatation and increased tubular cast area, which are age-dependent renal lesions associated with *UMOD* overexpression, might lead to identification of counteracting alleles for *UMOD*. Further, the quantification of fibrosis could lead to identification of additional risk alleles or counteracting alleles for *SHROOM3*. In addition, this new cohort could be a nice supplement to previous mouse studies. Higher resolution mapping could make it possible to identify the causative variant in *Esrrg* or counteracting alleles for *Far2* and *Wisp2*.

## Conclusion

To identify genes involved in renal aging by genome association studies, it is important to perform accurate phenotyping. The use of estimated glomerular filtration rate is not the optimal parameter for this purpose, as these formulas are unreliable and not validated in the elderly. Albuminuria can be used, as it might be caused by hyperfiltration due to age-related changes of the glomeruli, but it is important to correct for potential confounders such as hypertension and diabetes mellitus in a study population. In our opinion, the best phenotypes to examine in genetic analyses of renal aging should not be far downstream of the disease cascade; for example, examining nephrosclerotic changes in the early stage of their evolution and in the absence of confounding kidney disease could make detection of subtle changes possible even before they actually effect kidney function.

Mouse and other animal models such as rat and zebra fish can be used for follow-up of human GWAS for functional analysis of associated genes, of which several very promising examples are discussed in this article. Another advantage of animal models is that obtaining kidney tissue in a large human population for histological evaluation is difficult. Mouse models are time- and cost-effective while enabling control of environmental and population factors and are therefore very useful for identifying specific genetic variants based on histological age-related changes. Furthermore, the use of high-resolution genetic mapping tools, such as the DO mice and CC lines, will facilitate identification of the causative variants. Finally, when investigators identify genes associated with renal aging, they should determine whether the genes are specific for the kidney or are involved in a more general aging process. Animal models will make it possible to study the involvement of candidate genes in other organs.

Combining the power of human and animal studies can help us unravel the process of renal aging and perhaps even make personalized medicine possible in the future by performing risk-factor assessment to predict which individuals are susceptible for accelerated kidney aging and to develop preemptive therapy strategies.
